# Transcranial electrical brain stimulation modulates neuronal tuning curves in perception of numerosity and duration

**DOI:** 10.1016/j.neuroimage.2014.08.016

**Published:** 2014-11-15

**Authors:** Amir Homayoun Javadi, Iva K. Brunec, Vincent Walsh, Will D. Penny, Hugo J. Spiers

**Affiliations:** a Institute of Behavioural Neuroscience, Department of Experimental Psychology, University College London, WC1H 0AP, London, UK; b Institute of Cognitive Neuroscience, University College London, 17 Queen Square, WC1N 3AR London, UK; c Wellcome Trust Centre for Neuroimaging, University College London, 12 Queen Square, WC1N 3BG London, UK

**Keywords:** Receptive field, Neuronal tuning curve, Magnitude judgement, Numerosity, Duration, Time, Computational modelling

## Abstract

Transcranial direct current stimulation (tDCS) is a non-invasive brain stimulation method with many putative applications and reported to effectively modulate behaviour. However, its effects have yet to be considered at a computational level. To address this we modelled the tuning curves underlying the behavioural effects of stimulation in a perceptual task. Participants judged which of the two serially presented images contained more items (*numerosity judgement task*) or was presented longer (*duration judgement task*). During presentation of the second image their posterior parietal cortices (PPCs) were stimulated bilaterally with opposite polarities for 1.6 s. We also examined the impact of three stimulation conditions on behaviour: anodal right-PPC and cathodal left-PPC (*rA-lC*), reverse order (*lA-rC*) and *no-stimulation condition*. Behavioural results showed that participants were more accurate in numerosity and duration judgement tasks when they were stimulated with lA-rC and rA-lC stimulation conditions respectively. Simultaneously, a decrease in performance on numerosity and duration judgement tasks was observed when the stimulation condition favoured the other task. Thus, our results revealed a double-dissociation of laterality and task. Importantly, we were able to model the effects of stimulation on behaviour. Our computational modelling showed that participants' superior performance was attributable to a narrower tuning curve — smaller standard deviation of detection noise. We believe that this approach may prove useful in understanding the impact of brain stimulation on other cognitive domains.

## Highlights

• Behavioural effects of transcranial electrical stimulation were modelled.
• Computational modelling was based on tuning curves found in human and primates.
• Superior performance was attributable to a narrower tuning curve and vice versa.
• Results revealed a double-dissociation of laterality of stimulation and task.
• While stimulation improved performance in one task, it impaired performance in another.

## Introduction

Transcranial electrical brain stimulation has been claimed to be effective in the modulation of behaviour in many different applications; e.g. working memory (Fregni et al., 2005, Ohn et al., 2008), long-term memory (Javadi and Cheng, 2013, Javadi and Walsh, 2012, Javadi et al., 2012), motor tasks (Waters-Metenier et al., 2014, Zhou et al., 2014) as well as many clinical applications (da Silva et al., 2013, Fregni et al., 2005, Hummel et al., 2005), for review see (Madhavan and Shah, 2012, Nitsche and Paulus, 2011).

While such behavioural changes have been reported, the mechanisms underlying their responses are yet to be explored. To address this we created a computational model of the behavioural effects of tDCS stimulation of the left and right PPCs on neuronal tuning curves in numerosity processing and duration judgements. Although not conclusive, there is some evidence showing lateralisation of numerosity and duration judgement tasks (Cohen Kadosh et al., 2010, Dormal et al., 2008, Hauser et al., 2013, Vicario et al., 2013). Therefore we expected to see differential effects of stimulation based on laterality. This would have given us the chance to validate our model for different conditions.

Neurons tuned to numerosity were found in the macaque prefrontal and parietal cortices (Nieder and Miller, 2003, Nieder and Miller, 2004). In line with these findings, Piazza et al. (2004) conducted an fMRI adaptation study which showed evidence for systematic modulation of magnitude processing in the parietal cortex of humans. Participants were required to judge the number of dots on a screen after being habituated to either 16 or 32 dots. Their responses followed a U-shaped tuning curve which indicated an internalised numerical scale centred on the habituation number. We hypothesised that the effects of brain stimulation found in past studies can therefore be explained using the concept of tuning curves: Higher accuracy and decreased variance in behaviour following brain stimulation (Hauser et al., 2013, Vicario et al., 2013) can be explained by narrower tuning curves.

## Methods

### Participants

28 participants took part in this study. They were randomly assigned to one of the two tasks: the numerosity or the duration judgement task. Three participants were excluded from the analysis, either due to poor performance (n = 2), or due to displacement of electrodes (n = 1) leading to n = 12 for numerosity judgement task (7 females, age 22.80 ± 2.80) and n = 13 for duration judgement task (7 females, age 22.18 ± 2.18). All participants were healthy with no history of neurological or psychiatric disorders, had normal or corrected-to-normal vision and were naive to the purpose of the study. All were right-handed with a laterality quotient of at least 50 on the Edinburgh Handedness Inventory (Oldfield, 1971). All participants gave their written informed consent in accordance with the Declaration of Helsinki and the guidelines approved by the ethical committee of University College London (UCL).

### Apparatus

Experiments were run on desktop computers with a 17-inch CRT monitor and 100 Hz refresh rate with the resolution 1024 × 768 pixels. The monitor was 53 cm from the participants' eyes. Stimuli presentation and response time recording were achieved using MATLAB (v7.5; MathWorks Company) and the Psychtoolbox v3 (Brainard, 1997, Pelli, 1997). Data analyses were performed using SPSS (v20.0; LEAD Technologies, Inc.). Responses were made on a conventional computer keyboard using the index and middle fingers of the right hand.

### Procedure

**Fig. 1:**
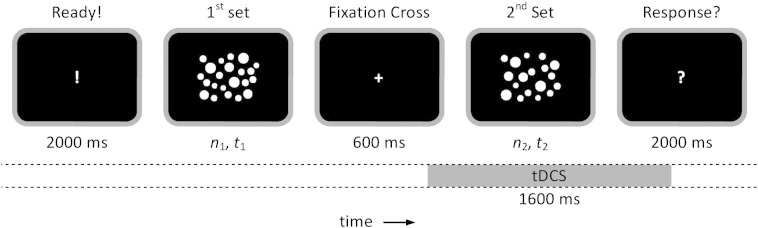
Procedure of the experiment for both numerosity and duration judgement tasks. For the numerosity judgement task, the number of dots varied between the two sets (*n* = {30, 32, 34, 36, 38}) but they were presented for the same duration (*t* = 1000 ms). For the duration judgement task, the number of dots was kept constant (*n* = 34) but the duration of presentation of each set changed (*t* = {800 ms, 900 ms, 1000 ms, 1100 ms, 1200 ms}). The diameter of dots was controlled in such a way that the overall covered surface was constant between the two sets. Stimulation was initiated 100 ms before the onset of the second set.

The experiment adopted a mixed-design with stimulation condition (3 conditions, see below) as within-subjects factor and task (Numerosity/Duration) as between-subjects factor. Two sets of dots were presented in a virtual 800 × 600 rectangle (28.93° × 21.69° visual angle). Participants were asked to judge which of the two sets contained more dots (numerosity judgement task) or which of the two sets was presented longer (duration judgement task). The numerosity of dots and duration of presentation of dots varied between the trials depending on the task. In the numerosity judgement task, durations of presentation of the two sets were identical, while the number of dots changed. In the duration judgement task, the two sets contained equal numbers of dots but were presented with varying durations. The diameter of dots was adapted pseudo-randomly to achieve a similar overall covered area to avoid possible confounds such as luminance and space (minimum and maximum diameter of 39 and 61 pixels equivalent to 1.44° and 2.25° visual degrees, respectively) (Fig. 1).

The experiment was split over 6 blocks with 30 s rest after each block. Each block contained 50 trials plus 10 training trials at the beginning of the first block.

### Transcranial direct current stimulation (tDCS)

Direct electrical current was administered using a neuroConn DC Brain Stimulator Plus unit (Rogue Resolutions, Wales, UK). It was delivered bilaterally via a pair of saline-soaked surface sponge electrodes (both 35 × 35 mm^2^) onto the left and right PPCs (P3 and P4 based on 10–10 international system of electrode placement). In one condition, the anode electrode was placed over P3 and the cathode electrode was placed over P4 (*lA-rC* stimulation condition). In the second condition, the placement of the electrodes was reversed (*rA-lC* stimulation condition).

Stimulation was administered on a trial-by-trial basis. In each trial, there was either 1600 ms of stimulation (lA-rC and rA-lC stimulation conditions) or none (*no-stimulation* condition). The onset of the stimulation was 100 ms before the onset of the 2nd set of dots. A square wave form was used with 1.5 mA of amplitude (current density of 1.22 μA/mm^2^). The stimulation was delivered during only the 2nd set of dots in each trial. This was followed by at least 3100 ms of no stimulation until presentation of the 1st set of dots of the next trial. Nitsche and Paulus (2001a) showed that the effect of stimulation of motor cortex on motor evoked potentials (MEP) does not last beyond the duration of stimulation for stimulations shorter than 5 min. Additionally Javadi et al. (2012) showed that the effects of 1600 ms of stimulation do not last beyond the duration of the stimulation Thus we did not expect any lasting effect beyond 1600 ms of stimulation. This method of stimulation has been shown to be effective in modulation of declarative memory (Javadi et al., 2012) and has been shown to be safe for humans (Iyer et al., 2005, Poreisz et al., 2007). The order of stimulation conditions was randomised throughout the session. Participants were informed that they would be stimulated briefly in each trial. They were acquainted with the sensation of the stimulation prior to the beginning of the experiment. All participants reported that they could feel the stimulation and none of them reported any discomfort.

The placement of the electrodes was switched between the blocks to achieve both lA-rC and rA-lC stimulation conditions. The placement of the initial polarity was counterbalanced between participants.

### Modelling of tuning curves

Using computational modelling, we aimed to calculate the tuning curves for different stimulation conditions (No-Stimulation/lA-rC/rA-lC). Considering the short duration of brain stimulation used in this study, it is reasonable to assume that the effects of stimulation in the preceding trial did not last beyond the duration of stimulation, therefore did not affect the first, nor the second set of stimuli in the current trial (Nitsche and Paulus, 2001b). Our modelling approach additionally made two assumptions: (1) the first and second sets of items in the no-stimulation condition contain the same level of detection noise. (2) The effect of stimulation is to perturb the representation of magnitude differences (rather than absolute values); i.e. trials with *n*_1_ = 30 and *n*_2_ = 32 evoke the same response as *n*_1_ = 36 and *n*_2_ = 38. On the basis of this assumption we considered a linear interaction of detection noise for the first and second sets of dots. While the width of the tuning curves associated with the judgement of magnitudes has been shown to be logarithmic when magnitudes change with an order of 4 (Piazza et al., 2004), due to the small increments by which our magnitudes change (minimum 30 to maximum 38 dots, shortest 800 ms to longest 1200 ms) it is appropriate to assume a linear interaction in this study.

We model the representation of magnitude differences with Gaussian population tuning curves

(1) $$p\left(x;\mu ,\sigma^{2}\right)=\frac{1}{\sigma \sqrt{2\pi }}\exp\left(-\frac{{\left(x-\mu \right)}^{2}}{2\sigma^{2}}\right)$$

in which *x* refers to either numerosity and duration judgement tasks.

**Fig. 2:**
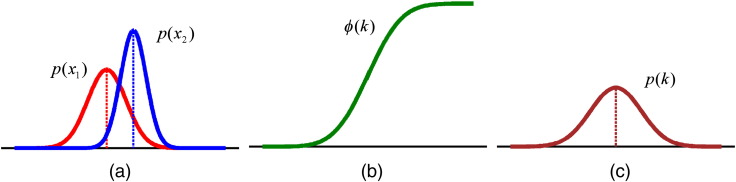
Procedure of action of tuning curves (a) on performance (b) and precision of perception (c). (a) shows two tuning curves, Eq. (1), corresponding to two sets of stimuli. (b) represents the cumulative normal distribution, Eq. (2), fitted to participant's performance. (c) demonstrates the resultant distribution, Eq. (5).

Fig. 2a shows the tuning curves for the first (red curve) and second (blue curve) sets in a given trial with means (*μ*_1_ and *μ*_2_) and standard deviations (${\sigma_{1}}^{2}$ and ${\sigma_{2}}^{2}$). Here, the representation during the second set has greater precision than the first (narrower width, ${\sigma_{1}}^{2}>{\sigma_{2}}^{2}$).

Having two tuning curves, *p*(*x*_1_) and *p*(*x*_2_), corresponding to the first and second sets respectively, one can derive the probability distribution of the difference, *p*(*k*), where *k* = *x*_2_ − *x*_1_. Here, *n* and *t* represent numerosity and duration of presentation of each set (Javadi and Aichelburg, 2012, Javadi and Aichelburg, 2013) and *k* = *n*_2_ − *n*_1_ for the numerosity judgement task and *k* = *t*_2_ − *t*_1_ for the duration judgement task, with *k* representing 9 levels of difference ([− 4 … + 4]).

Given that *p*(*x*_1_) and *p*(*x*_2_) are Gaussian, the distribution of the difference will also be Gaussian (see below). A participant's response will then follow the cumulative normal distribution

(2) $$\phi \left(k;\mu ,\sigma^{2}\right)=\frac{\sigma }{\sqrt{2\pi }}\int_{-\infty }^{x}\exp\left(-\frac{-\sigma^{2}{\left(x-\mu \right)}^{2}}{2}\right).$$

This equation is the psychometric function that has been used to fit behavioural data for two alternative forced choice tasks (Green and Swets, 1966). In the following, we fit this psychometric function to participants' responses (percentage of selection of the second set), as shown in Fig. 2b. Subsequently *ϕ*(*k*) was used to construct a normal distribution, *p*(*k*) (Fig. 2c), using parameters *μ* and *σ*^2^.

Given Gaussian distributions of magnitudes

(3) $$x_{1}\sim p\left(x_{1};\mu_{1},{\sigma_{1}}^{2}\right)$$

(4) $$x_{2}\sim p\left(x_{2};\mu_{2},{\sigma_{2}}^{2}\right)$$

the density over the difference is also Gaussian (Wackerly et al., 2008)

(5) $$k\sim p\left(k;\mu_{2}-\mu_{1},{\sigma_{1}}^{2}+{\sigma_{2}}^{2}\right).$$

With regard to the design of this study in which there is no-stimulation during the first set and three stimulation conditions during the second set, we expect four distributions possessing ${\sigma_{1}}^{2}$ and ${\sigma_{2,c}}^{2}$ in which *c* = 1, *c* = 2 and *c* = 3 represent no-stimulation, lA-rC and rA-lC stimulation conditions, respectively. Based on the first assumption

(6) $${\sigma_{2,1}}^{2}={\sigma_{1}}^{2}.$$

Similarly, having the ${\sigma_{\mathrm{fitted},1}}^{2}$ of the fitted curve *ϕ*(*k*; *μ*, *σ*^2^) to the psychometric function of no-stimulation condition and Eqs. (5), (6), the ${\sigma_{2,1}}^{2}$ would be

(7) $$\begin{array}{l} {\sigma_{\mathrm{fitted},1}}^{2}={\sigma_{1}}^{2}+{\sigma_{2,1}}^{2}\\ ∴{\sigma_{2,1}}^{2}={\sigma_{1}}^{2}={\sigma_{\mathrm{fitted},1}}^{2}/2. \end{array}$$

Similarly, using Eqs. (5), (7),

(8) $$∴{\sigma_{2,2}}^{2}={\sigma_{\mathrm{fitted},2}}^{2}-{\sigma_{1}}^{2}$$

(9) $$∴{\sigma_{2,3}}^{2}={\sigma_{\mathrm{fitted},3}}^{2}-{\sigma_{1}}^{2}.$$

### Statistical analysis

Performance and response times were recorded for analysis. *Performance* was calculated as the percentage of trials in which the second set was selected as the more numerous or longer set in numerosity and duration judgement tasks respectively. Trials with no response and with response time greater than 2.2 inter-quartile-range (IQR) above 75th percentile and smaller than 2.2 IQR below 25th percentile were removed from analysis (Hoaglin and Iglewicz, 1987). A psychometric function based on a cumulative normal distribution, Eq. (2), was fitted to the performance using the Palamedes toolbox (v1.4.4) for MATLAB (Prins and Kingdom, 2009). *σ*^2^ and *μ* were separately subjected to a mixed-factor analysis of variance (ANOVA) with stimulation condition (No-Stimulation/lA-rC/rA-lC) as a within subjects factor and task (Numerosity/Duration) as a between subjects factor. Subsequently, Bonferroni corrected post-hoc paired-sample *t*-tests were run to investigate the difference between different stimulation conditions. Goodness of fit was calculated using the Palamedes toolbox (Wichmann and Hill, 2001). These values were subjected to a mixed-factor analysis of variance (ANOVA) with stimulation condition (No-Stimulation/lA-rC/rA-lC) as a within subjects factor and task (Numerosity/Duration) as a between subjects factor. In order to ensure that there is no significant difference between goodness of fit in different stimulation conditions, we ran post-hoc paired-sample *t*-tests.

Response times were also analysed. Response times for trials with *k* < 0 and *k* > 0 were collapsed to achieve two groups of response times. A mixed-factor ANOVA with group (*k* < 0 and *k* > 0) and stimulation condition (No-Stimulation/lA-rC/rA-lC) as within subjects factors and task (Numerosity/Duration) as a between subjects factor was conducted. Subsequently post-hoc paired-sample *t*-tests were run to investigate the difference between groups of response times and tasks. The data were tested for normality of distribution. Effect sizes of partial Eta squared (*η_p_*^2^) are reported for the ANOVA. This measure indicates the proportion of variance in the dependent variable explained by the independent variable and is a value between 0 and 1.

## Results

**Fig. 3:**
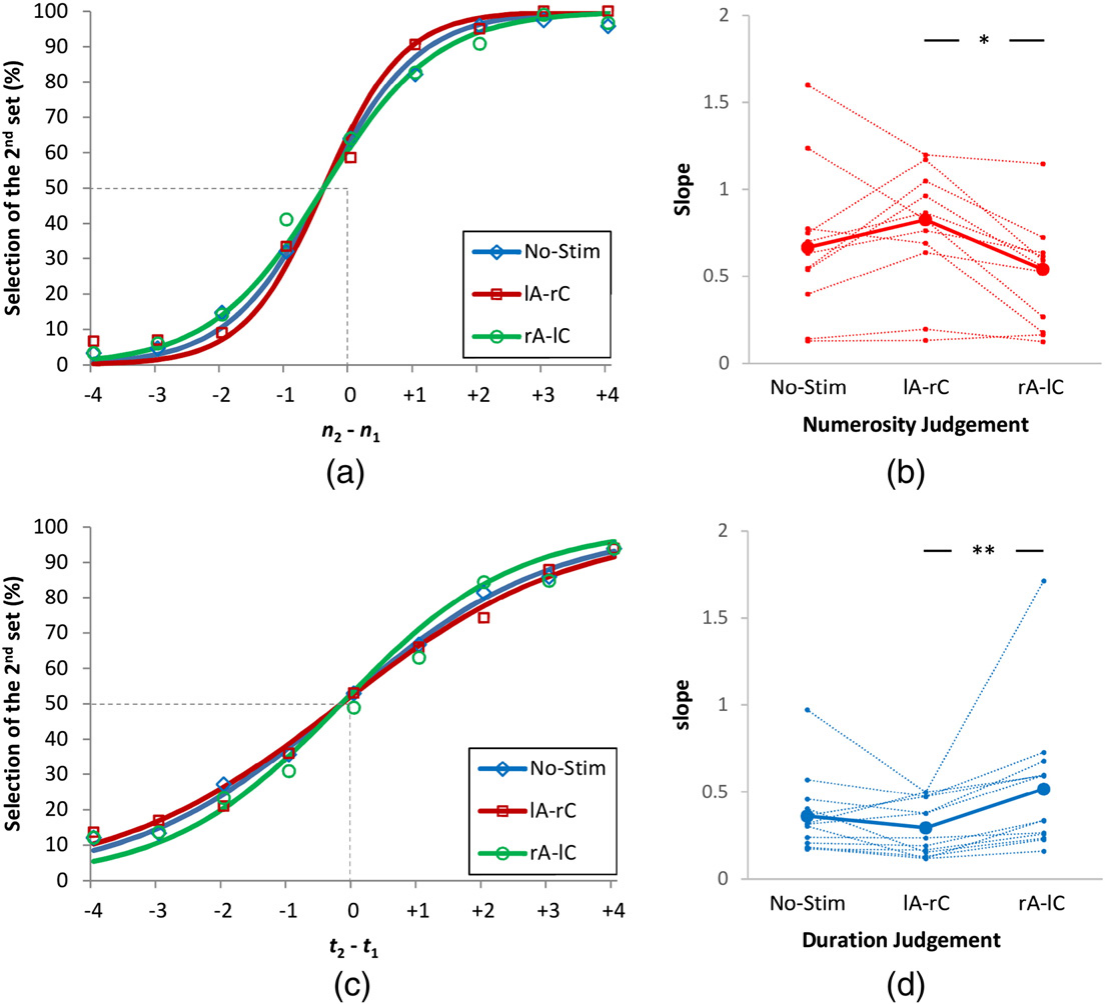
Psychometric functions of performance, defined as percentage of selection of the 2nd set, for (a) numerosity and (c) duration judgement task for different stimulation conditions. (b & d) Corresponding slopes of the fitted psychometric functions. They include individual data along with their median. *lA-rC* refers to the placement of an anode electrode on the left-PPC and a cathode on the right-PPC. *rA-lC* refers to the placement of an anode electrode on the right-PPC and a cathode on the left-PPC. *No-Stim* refers to no-stimulation condition. Error bars indicate one standard error of mean. *p < 0.05 and **p < 0.01 (Bonferroni corrected).

A total of 4.9% of trials were excluded from analysis comprising trials with no response and response time outliers. Psychometric functions indicating the mean of performance of participants for each stimulation condition are shown in Fig. 3.

Values for goodness of fit were subjected to a mixed-factor ANOVA. This test showed a non-significant effect of stimulation condition (No-Stim mean (SD): 0.558 (0.226), lA-rC: 0.542 (0.226), rA-lC: 0.577 (0.195), *F*(2, 46) = 0.651, *p* = 0.526, *η_p_*^2^ = 0.028), a non-significant effect of group (numerosity: 0.484 (0.183), duration: 0.629 (0. 183), *F*(1, 23) = 3.851, *p* = 0.062, *η_p_*^2^ = 0.143), and a non-significant interaction of factors (*F*(2, 46) = 3.897, *p* = 0.060, *η_p_*^2^ = 0.145). Post-hoc paired sample *t*-tests showed no significant difference for any comparison (*p*s > 0.137).

*σ*^2^ values were subjected to a mixed-factor ANOVA. This test showed a non-significant effect of stimulation condition (*F*(2, 46) = 0.696, *p* = 0.504, *η_p_*^2^ = 0.029), and a non-significant effect of group (*F*(1, 23) = 2.624, *p* = 0.119, *η_p_*^2^ = 0.102), but a significant interaction of factors (*F*(2, 46) = 14.652, *p* < 0.001, *η_p_*^2^ = 0.389). Bonferroni corrected post-hoc paired sample *t*-tests showed a significant difference between lA-rC and rA-lC stimulation conditions for both numerosity (*t*(11) = 3.272, *p* = 0.021) and duration (*t*(12) = − 4.493, *p* = 0.003) judgement tasks. No other comparisons were significant (*p*s > 0.108). *σ*^2^ values for different stimulation conditions and tasks are shown in Fig. 3. *μ* values were also subjected to a mixed-factor ANOVA. None of the comparisons were significant (*p*s > 0.178).

**Fig. 4:**
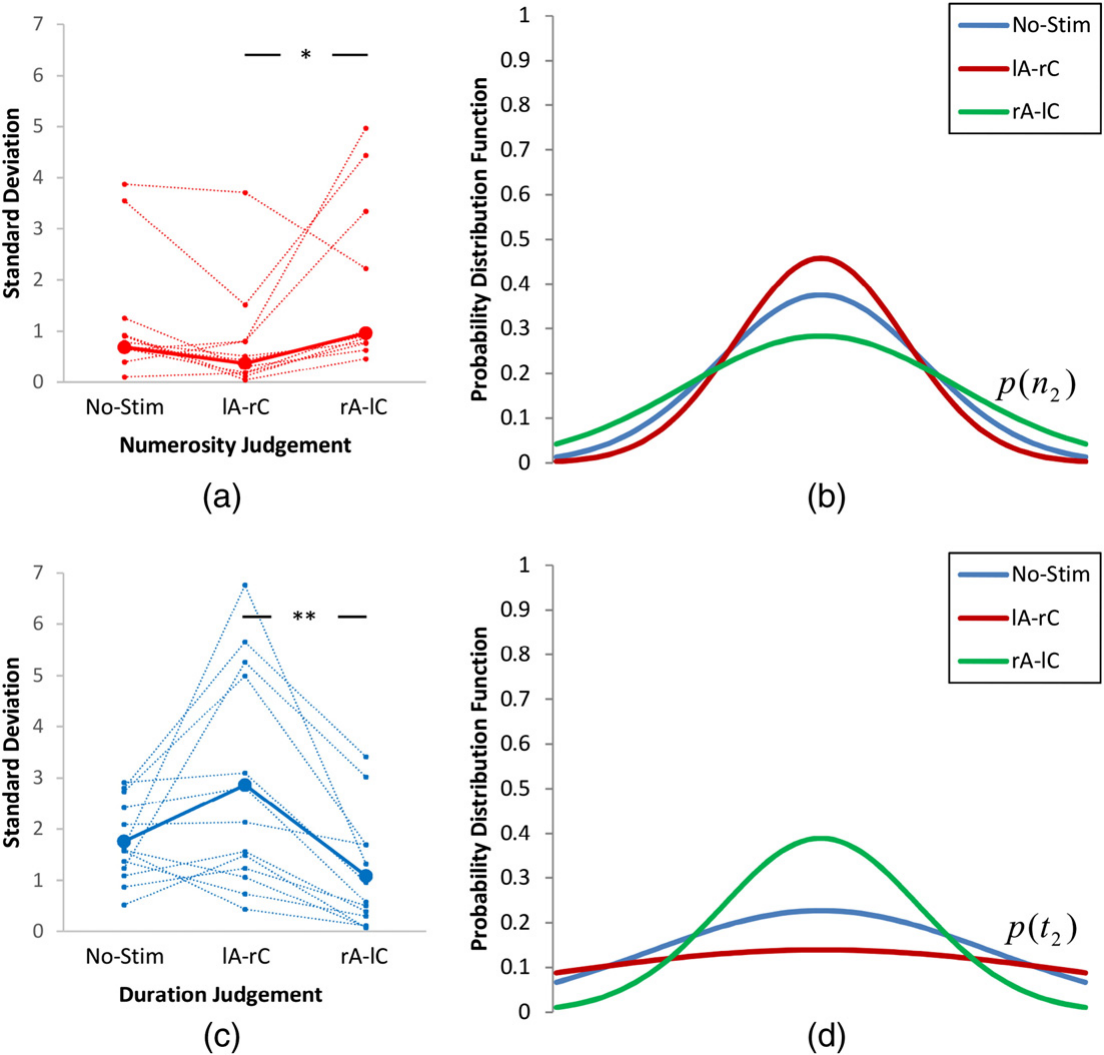
Standard deviation (a & c) of the original normal distributions that construct the psychometric functions shown in Fig. 3 and their corresponding distributions (b & d) for different stimulation conditions. (a & c) includes individual data along with their median. Standard deviation is *σ* as indicated in Eq. (1). Error bars indicate one standard error of mean. *p < 0.05 and **p < 0.01 (Bonferroni corrected).

We then constructed tuning curves fitted to the cumulative normal distribution curves shown in Fig. 3. We used Eqs. (8), (9) to approximate the standard deviations of *σ*_2,*c*_ for numerosity (Fig. 4a) and duration judgement (Fig. 4c) tasks. Corresponding tuning curves are shown in Figs. 4b and d, respectively. Confirming our hypothesis, a narrower tuning curve was observed for the anodal stimulation of the left-PPC and cathodal stimulation of the right-PPC for numerosity judgement task and for anodal stimulation of the right-PPC and cathodal stimulation of the left-PPC for duration judgement task. Conversely, the modelling revealed opposite effects for cathodal stimulation.

**Fig. 5:**
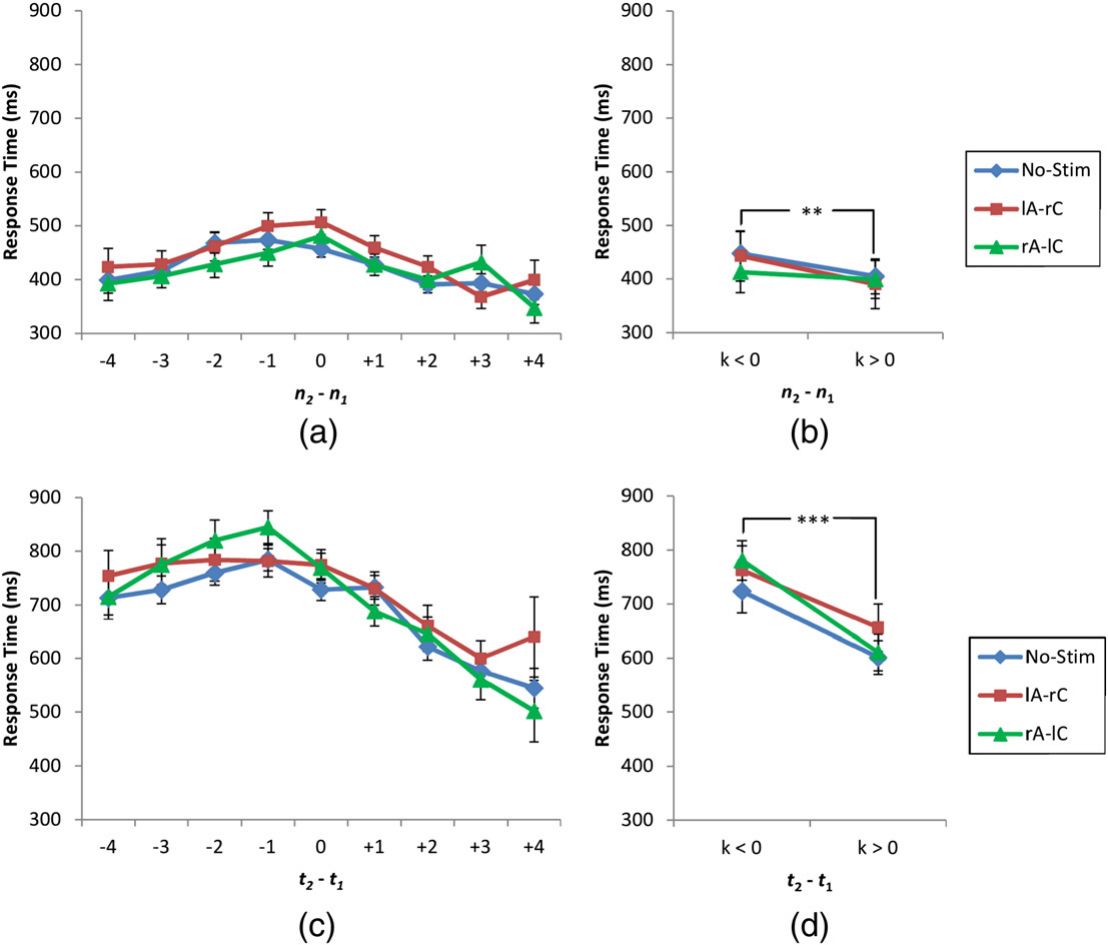
Response time (ms) for (a–b) numerosity judgement task and (c–d) duration judgement task. Error bars indicate one standard error of mean. **p < 0.01 and ***p < 0.001.

A mixed-factor ANOVA was run on response times. This test showed a significant effect of group of response times (response times collapsed over *k* < 0 and *k* > 0) (*F*(1, 23) = 39.007, *p* < 0.001, *η_p_*^2^ = 0.629), a significant interaction of group of response times and task (*F*(1, 23) = 12.752, *p* = 0.002, *η_p_*^2^ = 0.357), a significant effect of group (*F*(1, 23) = 29.973, *p* < 0.001, *η_p_*^2^ = 0.566), and a non-significant interaction of the three factors (*F*(2, 46) = 3.096, *p* = 0.055, *η_p_*^2^ = 0.119). No other effect was significant (*p*s > 0.132). Post-hoc paired-sample *t*-tests were run to investigate the difference between groups of response times in different tasks. These tests showed significant differences for numerosity judgement task (*t*(11) = 4.072, *p* = 0.002) and duration judgement task (*t*(11) = 5.465, *p* < 0.001). Fig. 5 shows response times for different levels of *k* and for collapsed groups. Thus, stimulation did not affect the response time.

## Discussion

By applying a computational modelling framework based on tuning curves found in human and primates (Nieder and Miller, 2003, Nieder and Miller, 2004, Piazza et al., 2004) we have found that higher performance was caused by a narrower tuning curve and vice versa. Furthermore, through the use of bilateral stimulation of the PPC with opposite polarities and a task that has been shown to be effective in studying reciprocal interaction of numerosity and duration (Javadi and Aichelburg, 2012, Javadi and Aichelburg, 2013) we showed a double-dissociation between left- and right-PPCs and task: application of anodal tDCS to the left-PPC and cathodal tDCS to the right-PPC (lA-rC) increased accuracy in the numerosity judgement task and impaired accuracy in the duration judgement task, while application of anodal tDCS to the right-PPC and cathodal tDCS to the left-PPC (rA-lC) increased accuracy in the duration judgement task and impaired accuracy in the numerosity judgement task.

Ma et al. (2006) proposed that sharper probability distribution functions (PDFs) are represented with higher neuronal firing rates. The psychometric functions shown in Fig. 3 are the result of interaction of two tuning curves as shown in Fig. 2a. Our short duration of stimulation along with our computational modelling approach enabled us to successfully disentangle contribution of the two tuning curves as shown in Fig. 4. This may be consistent with the excitatory and inhibitory effects of tDCS (Nitsche and Paulus, 2001b) and with our findings showing anodal stimulation of the left- and right-PPCs leading to higher precision of numerical and duration processing respectively.

The bilateral intraparietal sulci (IPS) and their surrounding areas have been implicated in the processing of numerosity across the visual and auditory dimensions (Piazza et al., 2006) and in numerosity and numerical symbols (Piazza et al., 2007). An fMRI study by Dormal et al. (2010) found that while simultaneous numerosity processing (arrays of dots) induced bilateral IPS activation, the right IPS was more activated in the processing of sequential stimuli. The bilateral activation of the PPC found in numerosity estimation was also observed during length and duration discrimination (Pinel et al., 2004). Neuroimaging studies of temporal estimation found activation in a broad fronto-parietal network with a right hemispheric dominance (Lewis and Miall, 2003). A meta-analysis of fMRI studies provided evidence that the left hemisphere appeared to be more commonly activated during addition and subtraction, and the right hemisphere during multiplication (Arsalidou and Taylor, 2011). As in some studies a bilateral activation has been found, we targeted both left- and right-PPCs with opposite polarities with the intention to enhance one hemisphere and suppress another highlighting the laterality effect of the tasks (although this is only an assumption and has not been shown physiologically). This procedure gave us the possibility to investigate importance of laterality of stimulation in the two numerosity and duration judgement tasks.

Laterality effects in the PPC were found by Vicario et al. (2013) following tDCS application. While cathodal stimulation of the right PPC led to overestimation, cathodal stimulation of the left PPC reduced the variability in reproducing time intervals. They found no effects of anodal stimulation. The disruptions observed following cathodal stimulation of the PPC can therefore be taken as evidence that different subsets of this area are involved in the processing of different components of time and estimation of its magnitude. Cohen Kadosh et al. (2010) found that anodal tDCS enhanced the acquisition of number symbols in a number comparison task when applied to the right, but not left side. In contrast, a recent study by Hauser et al. (2013) found that left anodal tDCS significantly enhanced performance in a number comparison and simple arithmetic tasks, while bilateral and right anodal tDCS did not induce any improvements. The authors argued that the left PPC in particular appears to be causally involved in numerosity processing. A degree of hemispheric asymmetry was reported in the contribution to the precision of judgments, as activation in the left IPS was found to be more strongly correlated with exact numerical judgments, while activation in the right IPS correlated more strongly with approximate judgments (Piazza et al., 2006). Dormal et al. (2008) applied rTMS to the IPS bilaterally and found that participants' performance in a numerosity judgement task was slowed down only after the left IPS was stimulated. Furthermore, a study using tDCS reports that stimulation of the left PPC was required to disrupt more precise discrimination of numbers close together, while bilateral stimulation was necessary to impair the discrimination of numbers further apart (Andres et al., 2005). While our design does not allow us to have a discrete conclusion regarding contribution of each laterality in the two tasks, our results suggest a bidirectional influence of PPC stimulation. In the numerosity judgement task, an improvement in performance was observed following lA-rC stimulation, while rA-lC stimulation impaired performance. Conversely, in the duration judgement task, an improvement in performance was observed following rA-lC stimulation, while lA-rC stimulation dampened it. This bidirectional influence is a novel finding which has not been reported in previous studies.

Electrical brain stimulation affects an area larger than the surface underneath the electrodes. These effects are modelled in various studies such as those by Bikson et al., 2012a, Bikson et al., 2012b. They showed in normal head models although the electrical current is spread over a wide area, the majority of the current is focused under the electrodes. Additionally we acknowledge that the effects of electrical brain stimulation do not limit to the brain area underneath the electrodes not only due to distribution of electrical current, but also by interaction of interconnected brain areas and modulation of brain networks as well. Nevertheless, our results show that posterior parietal cortices have a critical rule in perception of magnitude.

Our findings also agree with ATOM (A Theory Of Magnitude), which posits the parietal cortex as the primary site of magnitude judgements in different dimensions (Walsh, 2003a, Walsh, 2003b). This theorem was proposed to account for common neural processing of magnitudes in the different dimensions of time, space, and quantity. The proposed site of this processing is the parietal cortex where interacting and occasionally interfering inputs such as size, duration, and number are processed in terms of a common metric for action which can provide judgments or motor outputs relating to their magnitudes. However, the present results further the primary premise by providing evidence for functional specialisation within the parietal area.

Future work in this area should aim to achieve a more exact mapping of the brain areas responsible for numerosity and duration judgements. Overcoming the difficulties of precise temporal and spatial resolution imposed by different brain stimulation techniques could provide some insight into the underlying mechanism and its fit in proposed models of magnitude processing, e.g. (Bueti and Walsh, 2009, Walsh, 2003a, Walsh, 2003b). In terms of computational modelling, while Bikson et al., 2012a, Bikson et al., 2012b have extensively investigated patterns of current flow across the brain based on different settings and protocols of stimulation, our method is the first step towards modelling the effects of stimulation on behaviour.

In conclusion, we were able to conceptualise the behavioural effects of tDCS as the modulation of tuning curves in the perception of numerosity and duration, which may prove useful when applied to tDCS studies. Contrary to general consensus that electrical stimulation changes the global firing rate of the neurons, our results showed that this modulation could be selective. Moreover, the present study provides evidence for a double-dissociation of duration and numerosity processing in the posterior parietal cortex.

## Authors' contribution

AHJ conceived and designed the experiment. AHJ wrote the code and ran the study. AHJ & WDP analysed the data. AHJ, IKB, WDP, VW & HJS wrote the paper.
